# Structural and Functional Brain Connectivity Uniquely Contribute to Episodic Memory Performance in Older Adults

**DOI:** 10.3389/fnagi.2022.951076

**Published:** 2022-07-12

**Authors:** Kylie H. Alm, Anja Soldan, Corinne Pettigrew, Andreia V. Faria, Xirui Hou, Hanzhang Lu, Abhay Moghekar, Susumu Mori, Marilyn Albert, Arnold Bakker

**Affiliations:** ^1^Department of Psychiatry and Behavioral Sciences, Johns Hopkins University School of Medicine, Baltimore, MD, United States; ^2^Department of Neurology, Johns Hopkins University School of Medicine, Baltimore, MD, United States; ^3^Department of Radiology, Johns Hopkins University School of Medicine, Baltimore, MD, United States

**Keywords:** multimodal neuroimaging, individual differences, diffusion tensor imaging, resting state functional connectivity, episodic memory

## Abstract

In this study, we examined the independent contributions of structural and functional connectivity markers to individual differences in episodic memory performance in 107 cognitively normal older adults from the BIOCARD study. Structural connectivity, defined by the diffusion tensor imaging (DTI) measure of radial diffusivity (RD), was obtained from two medial temporal lobe white matter tracts: the fornix and hippocampal cingulum, while functional connectivity markers were derived from network-based resting state functional magnetic resonance imaging (rsfMRI) of five large-scale brain networks: the control, default, limbic, dorsal attention, and salience/ventral attention networks. Hierarchical and stepwise linear regression methods were utilized to directly compare the relative contributions of the connectivity modalities to individual variability in a composite delayed episodic memory score, while also accounting for age, sex, cerebrospinal fluid (CSF) biomarkers of amyloid and tau pathology (i.e., Aβ_42_/Aβ_40_ and p-tau_181_), and gray matter volumes of the entorhinal cortex and hippocampus. Results revealed that fornix RD, hippocampal cingulum RD, and salience network functional connectivity were each significant independent predictors of memory performance, while CSF markers and gray matter volumes were not. Moreover, in the stepwise model, the addition of sex, fornix RD, hippocampal cingulum RD, and salience network functional connectivity each significantly improved the overall predictive value of the model. These findings demonstrate that both DTI and rsfMRI connectivity measures uniquely contributed to the model and that the combination of structural and functional connectivity markers best accounted for individual variability in episodic memory function in cognitively normal older adults.

## Introduction

Growing interest in using neuroimaging methods to map the human connectome has led to advances in methods that allow for *in vivo* examination of both structural and functional connectivity throughout the brain. Structural (i.e., physical, anatomical) connections are typically measured using diffusion-weighted imaging (DWI), which allows for the visualization of white matter pathways by quantifying water diffusion properties in different types of brain tissue. Compared to ventricles and gray matter regions, which have more unrestricted, or isotropic, diffusion properties, white matter tracts (made up of myelinated axons) typically have more restricted, or anisotropic, diffusion due to the presence of myelin sheaths (Tournier et al., 2011; Jones et al., 2013; Soares et al., 2013). Diffusion tensor imaging (DTI) is a modeling technique used to index the direction and degree of anisotropic diffusion throughout brain tissue in order to characterize the microstructural properties of the underlying white matter (Alexander et al., 2007, 2011; Jones et al., 2013; Teipel et al., 2016).

By contrast, functional connections are typically measured as co-occurring fluctuations in brain activation patterns across different brain regions using resting state functional magnetic resonance imaging (rsfMRI). This technique measures intrinsic correlations between fluctuations in the fMRI blood oxygenation level-dependent (BOLD) signal across disparate brain regions while participants are at rest, in the absence of any cognitive task (Biswal et al., 1995, 2010; Park and Friston, 2013; Teipel et al., 2016). Regions exhibiting higher correlations are said to be functionally connected. Since rsfMRI relies on temporal dependencies in the BOLD signal and not underlying anatomical connections, it is possible to have functionally connected regions in the absence of direct structural connections (Honey et al., 2009); however, it is generally agreed that structural and functional connectivity are somewhat correlated (Honey et al., 2009; Suárez et al., 2020).

A growing body of literature suggests that both structural and functional connectivity tend to decline during normal aging and across the Alzheimer’s disease (AD) spectrum (for reviews, see Ferreira and Busatto, 2013; Contreras et al., 2015; Teipel et al., 2016; Damoiseaux, 2017; Moody et al., 2021; Yu et al., 2021). These age- and disease-related changes in structural and functional connectivity have largely been investigated separately for each respective modality. Only recently have studies begun to utilize multimodal neuroimaging methods to simultaneously examine both connectivity modalities. Moreover, only a few studies have examined multimodal structural and functional connectivity markers and their relation to cognition, particularly episodic memory. In terms of global cognition, Palesi et al. (2016) found that when combined functional and structural connectivity graphs were generated using DTI measures as weights for the functional connection metrics (i.e., edges), poorer cognition in mild cognitive impairment (MCI) and AD dementia patients, as measured by the Mini-Mental State Exam (MMSE), was associated with reductions in functional network measures (e.g., global efficiency, local efficiency, and connectivity strength). Similar results were obtained for memory performance, assessed with logical memory and spatial ability, as measured by the Rey-Osterrieth Complex Figure copy test. A recent study (Yu et al., 2020) also found that combined structural and functional features from connectomes both independently contributed to the prediction of MMSE scores and a list learning task (the Rey Auditory Verbal Learning Test, RAVLT) in MCI patients. In older adults with normal cognition at baseline, a longitudinal investigation using stepwise regression revealed that both structural connectivity change in the cingulum and caudate-cortical functional connectivity change uniquely contributed to the explained variance in memory changes over time, indexed by 5 minute delayed recall performance on the California Verbal Learning Test (Fjell et al., 2016). Similarly, structural and functional connectivity in parietal regions both independently accounted for 12-year changes in memory scores from the Free and Cued Selective Reminding Task in non-demented older adults (Edde et al., 2020).

The dearth of multimodal neuroimaging studies examining the relationship between the connectivity modalities and episodic memory has left open questions about the potential interplay between structural and functional connectivity in predicting individual differences in memory function in older adults. The present investigation sought to test whether examining the relative contributions of structural and functional neuroimaging connectivity measures together might improve the ability to predict individual differences in episodic memory among older adults. We utilized hierarchical and stepwise regression to test whether each modality provided independent, meaningful information in explaining the variance in memory performance. Our regression models also included cerebrospinal fluid (CSF) markers of amyloid (Aβ_42_/Aβ_40_) and phosphorylated tau (p-tau_181_), along with hippocampal and entorhinal gray matter volumes, which are recognized markers of AD-related pathology. We predicted that both structural and functional connectivity measures would uniquely contribute to individual variation in episodic memory performance in cognitively normal older adults, advocating for the utility of both connectivity modalities as important markers of individual differences in memory performance in older adults. In contrast, we predicted that the examined AD biomarkers would not contribute to individual differences in memory performance, based on our previous work (Alm et al., 2020) that found CSF markers of Aβ_42_ and total tau (t-tau), as well as medial temporal lobe gray matter volumes, were not significantly associated with delayed episodic memory performance in cognitively normal older adults. These measures were included, however, to test whether memory performance is better explained by the combination of structural and functional connectivity measures, after accounting for these AD biomarkers.

## Materials and Methods

### Study Design

Data in the current study were derived from a subset of participants enrolled in the BIOCARD study, an ongoing longitudinal prospective cohort study aimed at identifying early biomarkers of AD. As previously described (Albert et al., 2014), the BIOCARD study began at the intramural program of the Geriatric Psychiatry Branch of the National Institutes of Mental Health (NIMH) in 1995. Participants completed comprehensive neuropsychological and clinical assessments annually, including a physical and neurological examination, record of medication use, and behavioral and mood assessments. MRI scans, CSF samples, and blood specimens were collected approximately every 2 years. In 2005, the study was stopped for administrative reasons, and in 2009, it was re-initiated when a research team at Johns Hopkins University (JHU) re-established the cohort and resumed annual assessments. Participants again began to complete annual cognitive and clinical assessments. Biennial collection of MRI (including diffusion-weighted imaging and rsfMRI data), CSF, and amyloid PET data began in 2015. Collection of tau PET imaging data was initiated in 2020. The data included in the present investigation were acquired beginning in 2015, with longitudinal data collection ongoing.

### Participants

The original BIOCARD cohort was comprised of 349 participants, who enrolled in the study between 1995 and 2005. At baseline, all participants were judged to be cognitively normal, as ascertained by cognitive testing, and free of any significant medical problems [e.g., severe cardiovascular or cerebrovascular disease (CVD), chronic psychiatric disorders, or chronic neurologic disorders]. At the time of enrollment, participants were primarily middle-aged (*M* = 57.3, *SD* = 10.4, range = 20.0–85.8). By design, approximately 75% of the cohort had a first degree relative with AD dementia. Additional information regarding the BIOCARD cohort is detailed elsewhere (Albert et al., 2014).

The current study sample included cognitively normal participants with DTI, rsfMRI, CSF, and cognitive testing data available from the same visit. This yielded a sample of 107 participants (60.7% female) with a mean age of 69.01 (*SD* = 8.53, range = 34.43–89.08) and mean education of 17.50 years (*SD* = 2.09, range = 12–20). Participant characteristics are shown in Table 1.

**TABLE 1:** Participant characteristics.

Variable	Participants (*n* = 107)
Age in years, mean (SD)	69.01 (8.53)
Sex, females (%)	60.70%
Education, mean years (SD)	17.50 (2.09)
MMSE score, mean (SD)	29.33 (0.92)
CVLT long delay free recall, mean (SD)	14.25 (2.08)
LM delayed recall, mean (SD)	17.27 (3.08)

*MMSE, Mini-Mental State Examination; CVLT, California Verbal Learning Test; LM, Logical Memory.*

These data were collected between January 2015 and January 2017 as part of the ongoing longitudinal assessments of the larger BIOCARD cohort. All participants in the study sample were judged to be cognitively normal based on consensus diagnoses completed by the staff of the JHU BIOCARD Clinical Core, which is comprised of neurologists, neuropsychologists, research nurses, and research assistants. A syndromic diagnosis was first established (i.e., normal, MCI, Impaired not MCI, Dementia) based on three sources of information: (1) clinical data concerning the medical, neurological, and psychiatric status of the individual; (2) reports of changes in cognition by the participant and their informants; and (3) evidence of decline in cognitive performance based on review of longitudinal neuropsychological assessments of multiple cognitive domains with comparison to published norms. Participants were deemed free of other medical conditions that could affect cognitive function outside of the topic of study. In this study, the diagnosis of Impaired not MCI typically reflected contrasting results from the Clinical Dementia Rating (CDR) interview and the cognitive test scores, with the participant and informant expressing concerns about changes in cognition in daily life but no observed impairments on objective neuropsychological assessment or vice versa. Since participants with a diagnosis of Impaired not MCI (*n* = 20) do not meet criteria for MCI, they were included among the cognitively normal participants, consistent with prior publications (see Albert et al., 2014 for additional details). Finally, if a participant was determined to be not cognitively normal, then an etiologic diagnosis was made (e.g., AD, Frontotemporal Dementia, Lewy Body Dementia, etc.). This diagnostic approach is consistent with the guidelines of the National Institute on Aging – Alzheimer’s Association working groups (Albert et al., 2011; McKhann et al., 2011) and comparable to the approach employed at the National Institute on Aging Alzheimer’s Disease Centers program. All diagnoses were made without knowledge of the MRI or CSF biomarker measures.

### Image Acquisition

Magnetic resonance imaging scans were collected on a 3T Philips Achieva scanner (Eindhoven, Netherlands). Diffusion-weighted images were acquired using a spin-echo sequence (TR = 7454 ms, TE = 75 ms, FOV = 260 mm × 260 mm, 0.81 mm × 0.81 mm × 2.2 mm voxels, flip angle = 90°, *b*-value = 700, number of gradients = 33, 70 axial slices, 275 s scan duration). Resting state BOLD data were acquired using a gradient-echo sequence (TR = 3000 ms, TE = 30 ms, flip angle = 75°, FOV = 212 mm × 212 mm, 3.3 mm × 3.3 mm × 3.3 mm voxels, 48 axial slices, 420 s scan duration). T1-weighted structural images were also acquired using a magnetization-prepared rapid gradient-echo (MPRAGE) sequence for anatomical reference and image registration (TR = 6.8 ms, TE = 3.1 ms, shot interval = 3000 ms, inversion time = 843 ms, flip angle = 8°, FOV = 256 mm × 256 mm, 1 mm × 1 mm × 1.2 mm voxels, 170 sagittal slices, 359 s scan duration).

### Image Processing

#### Diffusion Imaging

Quality control and DTI reconstruction were performed using MRICloud (Mori et al., 2016)^1^, which follows the pipeline of DTIStudio (Jiang et al., 2006) for subject motion and eddy current correction, as well as tensor fitting. MRICloud offers a fully automated multi-atlas image parcellation algorithm, which combines the image transformation algorithm, Large Deformation Diffeomorphic Metric Mapping (LDDMM; Christensen et al., 1997; Miller et al., 1997; Grenander and Miller, 1998) based on complementary contrasts [mean diffusivity (MD), fractional anisotropy (FA), and fiber orientation; Ceritoglu et al., 2009], and a likelihood fusion algorithm for DTI multi-atlas mapping and parcellation (Tang et al., 2014). The DTI multi-atlas template set contains 12 healthy adult brains, and results in the parcellation of 168 brain structures, from which vectors of DTI scalar metrics (three eigenvalues) were extracted. Parcellations for each participant were visually inspected to ensure that the automated segmentation process yielded accurate delineations of the structures of interest.

Building on our previous study (Alm et al., 2020), we chose to focus our analyses on radial diffusivity (RD), as it was the most sensitive DTI measure in accounting for individual differences in episodic memory in our earlier findings. RD is an average of the two minor eigenvalues, reflecting diffusion perpendicular to the primary axis of diffusion. The absolute diffusivities, including RD, may be more sensitive to specific microstructural changes, compared to FA (Alexander et al., 2011). We also sought to restrict our number of *a priori* comparisons in order to minimize inflated false positive rates due to multiple comparisons. Given our prior findings and other established links between medial temporal lobe white matter tracts and episodic memory function, the fornix (restricted to the body and column due to resolution constraints) and hippocampal cingulum were selected as the structural connectivity measures of interest. Region of interest (ROI)-specific RD measures were obtained by averaging the left and right hemisphere RD values across all of the voxels within an ROI.

#### Resting State Functional Magnetic Resonance Imaging

Standard preprocessing of the resting state BOLD data was performed using SPM and in-house MATLAB scripts. Preprocessing steps included slice timing correction, realignment, normalization to MNI standard space, and spatial smoothing using a Gaussian filter with a full-width half-maximum of 4 mm (Hou et al., 2019). Data were detrended and bandpass-filtered to 0.01–0.1 Hz in order to retain low-frequency fluctuation components. Motion scrubbing was performed to discard volumes with a displacement of 0.5 mm or greater relative to the prior volume (Power et al., 2012, 2014). Volumes immediately before and after the displaced volumes were also discarded to account for temporal spread of artifactual signal resulting from the temporal filtering in the low-frequency functional signal (Chan et al., 2014).

Preprocessed images were then parcellated into 114 ROIs and grouped into 7 large-scale resting state functional connectivity networks based on the parcellation of Yeo et al. (2011). The functional connectivity networks included 5 cognitive networks: the executive control network, default mode network, limbic network, dorsal attention network, and salience/ventral attention network (henceforth referred to as the salience network); and 2 sensory-motor networks: the visual network and somatomotor network. The present study focused only on the 5 cognitive networks.

After regressing out nuisance covariates of whole brain signal, white matter signal, CSF signal, and six rigid-body motion parameters, cross-correlation coefficients were computed between all pairs of ROIs. Fisher-z transformations were performed in order to transform correlation coefficients into z-scores, yielding a 114 × 114 pairwise connectivity matrix. To calculate network-wise functional connectivity, the connectivity matrix was reduced from 114 × 114 to 7 × 7 by averaging the z-transformed correlations belonging to the same network.

#### Volumetric Magnetic Resonance Imaging

Gray matter volumes of interest included the volume of the entorhinal cortex and hippocampus. These ROIs were derived from the same MRICloud (see text footnote 1; Mori et al., 2016) multi-atlas parcellation methods described above. ROI volumes for the entorhinal cortex and hippocampus were measured by summing the number of voxels within each ROI and were averaged across hemispheres.

### Cerebrospinal Fluid Assessments

CSF was collected via lumbar puncture during the same visit as MRI acquisition. 20 ml CSF was collected in the morning between 8 and 10 am after an overnight fast into a 50 ml polypropylene tube. After mixing and centrifugation at 2000 rpm for 15 min, 500 μl aliquots of CSF were frozen at −80°C within 60 min of collection. CSF Aβ_42_ (picograms/ml), Aβ_40_ (picograms/ml), and p-tau_181_ (picograms/ml) were measured using the Lumipulse G1200 assay (Fujirebio, Malvern, PA, United States). The ratio of CSF Aβ_42_/Aβ_40_ and p-tau_181_ were used in the current analyses. Assays were run in duplicate, and all samples were run in a single batch. Intra-assay coefficient of variation for this assay was 3.4% for Aβ_42_, 2.7% for Aβ_40_, and 1.8% for p-tau_181_. Three participants did not have CSF values available for the visit corresponding to their neuroimaging data and were therefore treated as missing cases for the CSF variables in the regression analyses.

### Delayed Episodic Memory Composite Score

A delayed verbal episodic memory composite score was derived from performance on tasks within the neuropsychological battery administered during the same visit as MRI acquisition and lumbar punctures. Specifically, z-scored performance was computed for the California Verbal Learning Test (CVLT) long delay free recall and the Wechsler Memory Scale Logical Memory (LM) delayed recall measures. For each participant, the z-scored measures were then averaged to yield a single delayed memory composite score, as used in prior work in this cohort (Alm et al., 2020).

### Statistical Analyses

Statistical analyses were performed using SPSS (Version 27). Hierarchical linear regression was used to examine the relative contributions of structural and functional connectivity markers to individual differences in delayed episodic memory performance, above and beyond potential contributions of CSF markers and gray matter volumes.

For the hierarchical regression model, the dependent variable was the composite delayed episodic memory score, and independent variables were added in blocks based on the different categories of variables, with simultaneous variable entry for each block. Step 1 included demographic variables of age and sex (years of education was not included, because it was not found to be a significant predictor of delayed episodic memory performance in our prior work; Alm et al., 2020). In Step 2, the ratio of CSF Aβ_42_/Aβ_40_ and p-tau_181_ were added as separate independent variables. In Step 3, entorhinal and hippocampal volume measures were added as separate independent variables. In Step 4, DTI microstructural measures (i.e., mean RD values) were added as separate independent variables for the fornix and hippocampal cingulum. In Step 5, network functional connectivity measures were added as separate independent variables for each of the following networks: executive control, default mode, limbic, dorsal attention, and salience network.

Based on the results of this analysis, a secondary analysis was conducted using stepwise linear regression to assess which measures entered on their own, rather than in blocks based on the type of measure, best accounted for individual variation in episodic memory. Stepwise regression also allowed us to examine which combination of measures predicted the highest proportion of explained variance in delayed memory. Unlike hierarchical regression, in which a block of variables can be entered simultaneously in a user-determined order, stepwise regression utilizes a mathematically driven approach to order of entry, whereby an algorithm determines which set of variables maximizes the overall proportion of explained variance. Independent variables were added one at a time to the model and subsequently removed if they did not statistically improve the overall model. Again, the dependent variable was composite delayed episodic memory score. The independent variables included were age, sex, Aβ_42_/Aβ_40_, p-tau_181_, entorhinal volume, hippocampal volume, fornix RD, hippocampal cingulum RD, and salience network resting state functional connectivity.

## Results

### Hierarchical Linear Regression

In the hierarchical regression model, Step 1 included demographic variables of age and sex, [*R*^2^ = 0.09, *F*(2,101) = 5.15, *p* = 0.007] and revealed that sex was significantly associated with composite delayed memory score, with females having higher memory performance than males [β = 0.25, *t*(101) = 2.63, *p* = 0.01; Table 2]. Age was not a significant independent predictor of delayed memory performance [β = −0.17, *t*(101) = −1.74, *p* = 0.09]. The addition of the CSF markers in Step 2 did not yield a significant increase in the proportion of explained variance [*ΔR^2^* = 0.00, *ΔF*(2,99) = 0.18, *p* = 0.84], nor were Aβ_42_/Aβ_40_ or p-tau_181_ significant predictors of episodic memory [β = 0.06, *t*(99) = 0.50, *p* = 0.62 and β = 0.001, *t*(99) = 0.01, *p* = 0.99, respectively]. Sex remained a significant predictor [β = 0.25, *t*(99) = 2.54, *p* = 0.01], but age was not significantly associated with memory [β = −0.16, *t*(99) = −1.60, *p* = 0.11]. Similarly, the addition of gray matter volumes in Step 3 did not significantly improve the model [*ΔR^2^* = 0.01, *ΔF*(2,97) = 0.41, *p* = 0.66]. Age [β = −0.18, *t*(97) = −1.74, *p* = 0.09], sex [β = 0.21, *t*(97) = 1.89, *p* = 0.06], entorhinal volume [β = −0.05, *t*(97) = −0.46, *p* = 0.65], hippocampal volume [β = −0.08, *t*(97) = −0.71, *p* = 0.48], Aβ_42_/Aβ_40_ [β = 0.05, *t*(97) = 0.42, *p* = 0.67], and p-tau_181_ [β = −0.01, *t*(97) = −0.07, *p* = 0.94] were not significantly associated with memory in this model.

**TABLE 2:** Hierarchical regression explaining variability in delayed episodic memory composite.

Delayed memory composite score	Independent variables	β	*t*-value	*F*	*ΔF*	*R* ^2^	*ΔR^2^*
Step 1				5.15**		0.09	
	Age	–0.17	–1.74				
	Sex	0.25	2.63**				
Step 2				2.62*	0.18	0.09	0.00
	Age	–0.16	–1.60				
	Sex	0.25	2.54**				
	Aβ_42_/Aβ_40_	0.06	0.50				
	P-tau_181_	0.001	0.01				
Step 3				1.86	0.41	0.10	0.01
	Age	–0.18	–1.74				
	Sex	0.21	1.89^†^				
	Aβ_42_/Aβ_40_	0.05	0.42				
	P-tau_181_	–0.01	–0.07				
	Entorhinal volume	–0.05	–0.46				
	Hippocampal volume	–0.08	–0.71				
Step 4				2.94**	5.63**	0.20	0.10
	Age	0.02	0.17				
	Sex	0.23	2.16*				
	Aβ_42_/Aβ_40_	0.05	0.47				
	P-tau_181_	–0.05	–0.47				
	Entorhinal volume	–0.08	–0.73				
	Hippocampal volume	–0.06	–0.55				
	Fornix RD	–0.43	–2.93**				
	Hippocampal cingulum RD	0.31	2.76**				
Step 5				2.45**	1.54	0.26	0.06
	Age	0.04	0.32				
	Sex	0.22	1.91^†^				
	Aβ_42_/Aβ_40_	0.08	0.66				
	P-tau_181_	–0.04	–0.30				
	Entorhinal volume	–0.09	–0.84				
	Hippocampal volume	–0.11	–0.93				
	Fornix RD	–0.53	–3.41***				
	Hippocampal cingulum RD	0.34	2.99**				
	RS control	0.07	0.60				
	RS default	0.01	0.12				
	RS limbic	0.03	0.29				
	RS dorsal attention	0.13	1.23				
	RS salience	–0.28	–2.36*				

^†^p < 0.06; *p < 0.05; **p < 0.01; ***p < 0.001. RD, radial diffusivity; RS, resting state. Bolded values represent statistically significant findings.

**FIGURE 1:**
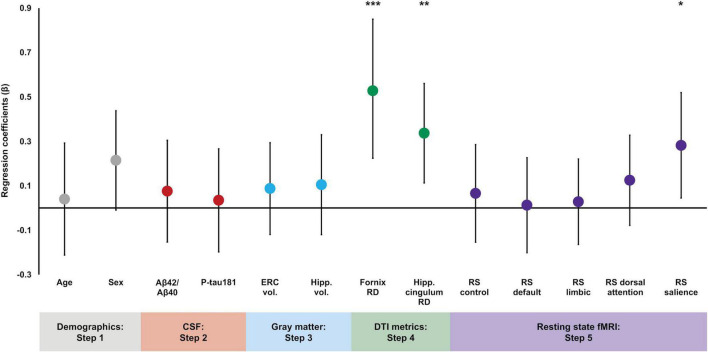
Regression coefficient betas (absolute values) from the hierarchical linear regression plotted for variables of interest color coded based on entry into the regression. Error bars are 95% confidence intervals. Demographic variables were entered in Step 1, CSF measures were entered in Step 2, gray matter volumes were entered in Step 3, DTI measures were entered in Step 4, and functional connectivity measures were entered in Step 5. Fornix RD, hippocampal cingulum RD, and salience network resting state connectivity were significant predictors of delayed episodic memory performance. *p < 0.05; **p < 0.01; ***p < 0.001.

By contrast, the addition of DTI microstructural measures in Step 4 significantly increased the proportion of variance explained in episodic memory performance [*ΔR^2^* = 0.10, *ΔF*(2,95) = 5.63, *p* = 0.005], and both fornix RD and hippocampal cingulum RD were significantly associated with episodic memory [β = −0.43, *t*(95) = −2.93, *p* = 0.004 and β = 0.31, *t*(95) = 2.76, *p* = 0.007, respectively]. Specifically, lower fornix RD and higher hippocampal cingulum RD were associated with better delayed memory performance. Sex was a significant predictor [β = 0.23, *t*(95) = 2.16, *p* = 0.03], but no other variables were significant (*p’s* > 0.47). Finally, there was no significant change in the proportion of explained variance after the addition of the resting state functional connectivity measures in Step 5 [*ΔR^2^* = 0.06, *ΔF*(5,90) = 1.54, *p* = 0.19]. However, resting state functional connectivity within the salience network was a significant predictor of episodic memory [β = −0.28, *t*(90) = −2.36, *p* = 0.02], such that lower resting state connectivity values were associated with better delayed memory performance. Fornix RD and hippocampal cingulum RD remained significant [β = −0.53, *t*(90) = −3.41, *p* = 0.001 and β = 0.34, *t*(90) = 2.99, *p* = 0.004, respectively], but none of the other resting state networks were significantly associated with memory performance and no other variables reached significance (*p’s* > 0.22; see Table 2). In order to visualize the magnitude of each variable’s contribution to the final regression model, standardized regression coefficients from Step 5 are plotted in Figure 1.

Additionally, we computed partial correlations, controlling for age and sex, to test for relationships between the 2 structural and 5 functional connectivity measures. There was a significant negative correlation between fornix RD and salience network functional connectivity [*r*(103) = −0.34, *p* < 0.001], indicating that decreased fornix RD was associated with increased salience network connectivity. There were no significant correlations between fornix RD and the other functional connectivity networks (absolute *r’s* < 0.15, *p’s* > 0.12) or between hippocampal cingulum RD and the functional connectivity networks (absolute *r’s* < 0.13, *p’s* > 0.19).

### Stepwise Linear Regression

To further explore the potential contributions of the salience network to individual variation in memory performance, a stepwise linear regression was conducted with the following predictors: age, sex, Aβ_42_/Aβ_40_, p-tau_181_, entorhinal volume, hippocampal volume, fornix RD, hippocampal cingulum RD, and salience network resting state functional connectivity. Results are presented in Table 3. Sex, fornix RD, hippocampal cingulum RD, and salience network functional connectivity were selected as significant predictors in the final model. Moreover, the addition of each one of the variables significantly improved the model. In Step 1, sex emerged as a significant predictor [*R*^2^ = 0.07, *F*(1,102) = 7.13, *p* = 0.01], followed by significant increases in the proportion of explained variance after the inclusion of fornix RD in Step 2 [*ΔR^2^* = 0.04, *ΔF*(1,101) = 4.83, *p* = 0.03], hippocampal cingulum RD in Step 3 [*ΔR^2^* = 0.07, *ΔF*(1,100) = 9.11, *p* = 0.003], and salience network functional connectivity in Step 4 [*ΔR^2^* = 0.04, *ΔF*(1,99) = 4.48, *p* = 0.04]. Therefore, while the strongest model included all four variables [*R*^2^ = 0.22, *F*(4,99) = 6.89, *p* < 0.001], each variable added unique predictive information to the model to improve the ability to explain variability in delayed episodic memory.

**TABLE 3:** Stepwise regression explaining variability in delayed episodic memory composite.

Delayed memory composite score	Independent variables	β	*t*-value	*F*	*ΔF*	*R* ^2^	*ΔR^2^*
Step 1				7.13**		0.07	
	Sex	0.26	2.67**				
Step 2				6.12**	4.83*	0.11	0.04
	Sex	0.23	2.48*				
	Fornix RD	–0.21	−2.20*				
Step 3				7.44***	9.11**	0.18	0.07
	Sex	0.26	2.84**				
	Fornix RD	–0.38	−3.54***				
	Hippocampal cingulum RD	0.33	3.02**				
Step 4				6.89***	4.48*	0.22	0.04
	Sex	0.26	2.91**				
	Fornix RD	–0.48	−4.16***				
	Hippocampal cingulum RD	0.35	3.29***				
	RS salience	–0.21	−2.12*				

Variables entered into model: age, sex, Aβ_42_/Aβ_40_, p-tau_181_, entorhinal volume, hippocampal volume, fornix RD, hippocampal cingulum RD, salience network functional connectivity. RD, radial diffusivity; RS, resting state. *p < 0.05; **p < 0.01; ***p < 0.001. Bolded values represent statistically significant findings.

**FIGURE 2:**
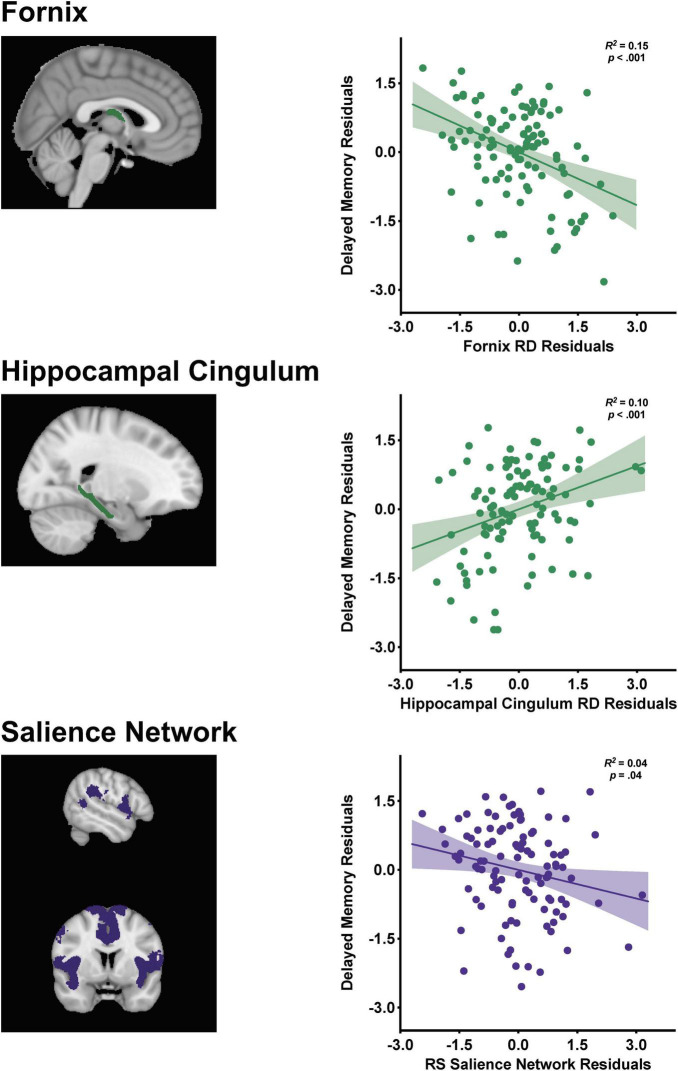
Visualization of the fornix [restricted to the body and column due to resolution constraints], hippocampal cingulum, and salience network with corresponding partial regression plots from the stepwise linear regression with standardized residuals depicting the relationships between the structural and functional connectivity markers with delayed episodic memory performance. Shaded 95% confidence interval bands.

Partial regression plots from the final stepwise regression model (Step 4) are displayed in Figure 2 depicting the relationships between the structural and functional connectivity measures with delayed memory. Memory performance was negatively associated with fornix RD [β = −0.48, *t*(99) = −4.16, *p* < 0.001] and salience network functional connectivity [β = −0.21, *t*(99) = −2.12, *p* = 0.04], such that decreased fornix RD and salience network connectivity were associated with better memory performance. There was a positive association between hippocampal cingulum RD and delayed memory [β = 0.35, *t*(99) = 3.29, *p* = 0.001], indicating that increased RD was related to better memory performance.

## Discussion

This study demonstrated that both structural and functional connectivity markers uniquely contributed to the explained variance in episodic memory performance in cognitively normal older adults. White matter microstructure in medial temporal lobe tracts of the fornix and hippocampal cingulum, as well as functional connectivity of the salience network were each independently associated with delayed memory. Additionally, a stepwise linear regression model found that the addition of each of these variables significantly improved the model, suggesting that the strongest model was one that included both structural and functional connectivity measures. Taken together, these findings indicate that a multimodal approach combining measures from both structural and functional connectivity modalities best accounts for individual differences in episodic memory among cognitively normal older adults.

These findings are in line with recent work demonstrating that both structural and functional connectivity measures independently contribute to explained variance in longitudinal memory changes in non-demented older adults (Fjell et al., 2016; Edde et al., 2020; Pur et al., 2022), as well as within a cross-sectional sample of MCI patients (Yu et al., 2020). These findings are also consistent with prior research suggesting that a multimodal approach combining features from structural and functional connectivity modalities is better than either modality alone in discriminating AD dementia patients from healthy controls (Schouten et al., 2016; but see Dyrba et al., 2015 where combining modalities did not improve prediction) and participants with subjective cognitive decline from healthy controls (Yan et al., 2019; Chen et al., 2022).

In contrast, CSF Aβ_42_/Aβ_40_, CSF p-tau_181_, and gray matter volumes of the entorhinal cortex and hippocampus were not significantly associated with variability in delayed memory performance in this cognitively normal sample. This was also the case in our previous study on the relationship between episodic memory and various brain biomarkers (Alm et al., 2020). CSF measurements reflect more global changes, while the significant associations in the present investigation were localized to particular regions or networks; therefore, it is possible that the global CSF measures were not sensitive enough to capture individual differences in memory performance, at least among cognitively normal individuals. Other cross-sectional studies have also reported no evidence of relationships between verbal memory tasks and CSF AD biomarkers among cognitively normal/non-demented participants (Schott et al., 2010; Li et al., 2014; Bos et al., 2018; Seo et al., 2021). Similarly, while gray matter volumes were localized to specific medial temporal lobe regions, these are considered measures of macrostructure, and may consequently be unable to detect subtle individual differences when compared to microstructural alterations of particular white matter tracts. It is possible that associations between memory and gray matter volume may emerge in samples that include participants with MCI or dementia, which are likely to exhibit more variability in gray matter volume measures.

Functional connectivity within the executive control, default mode, limbic, and dorsal attention resting state networks were not associated with delayed memory performance. Only the salience network demonstrated a significant association with delayed episodic memory performance. The limited findings within the resting state modality could be explained by previous research suggesting that alterations in structural connectivity seem to emerge earlier during aging than functional connectivity changes (Wang et al., 2018; Filippi et al., 2020). For example, Filippi et al. (2020) argued that the early changes in structural connectivity may be the mechanism that propagates later changes in large-scale functional connectivity networks. Therefore, in this cognitively normal sample, alterations in structural connectivity measures may appear stronger, and prior to, alterations in functional connectivity measures. It should be noted, however, that the opposite pattern of earlier changes in functional connectivity compared to structural connectivity has also been reported (Palesi et al., 2016; Chen et al., 2022), warranting further research to clarify the temporal time course of these connectivity changes. Similarly, when differentiating AD dementia patients from healthy controls, Schouten et al. (2016) found that stepwise classification was most improved by the addition of DTI features, and marginally improved when functional connectivity was further added. This parallels our stepwise regression findings, in which DTI measures of the fornix and hippocampal cingulum showed the largest improvement in our model. However, the results of this study do not provide a rationale for the salience network emerging as the sole significant predictor among the functional connectivity networks examined. It is possible that alterations in salience network connectivity occur earlier than in other networks, but this has not been previously reported, and future studies are needed to explore this possibility.

It has also been suggested that stronger coupling (i.e., correlation) between the structural and functional connectivity modalities may be maladaptive or represent pathological aging processes. For example, increased coupling between the modalities has been shown throughout progression along the AD spectrum (Palesi et al., 2016; Wang et al., 2018; Dai et al., 2019; Cao et al., 2020). Additionally, increased coupling has also been shown to be negatively correlated with memory performance (Wang et al., 2018; Edde et al., 2020), implying an adverse impact of strengthened structural and functional connectivity coupling on memory function. It is therefore possible that the relative lack of functional connectivity findings in this study could reflect a lack of structural-functional coupling within the ROIs examined here, among cognitively normal individuals, and that more pronounced functional connectivity effects could emerge over time. It is not possible to test this in a cross-sectional study, warranting future longitudinal investigations.

Interestingly, a significant negative correlation between fornix RD and salience network functional connectivity was observed after controlling for age and sex, but no correlations were found with the other functional networks. Past results in cognitively normal/non-demented samples have been inconsistent with respect to the direction of structural – functional connectivity relationships. Some studies have found positive correlations between the modalities in older adults, suggesting that higher white matter integrity is associated with higher functional connectivity (Andrews-Hanna et al., 2007; Chen et al., 2009; Teipel et al., 2010; Davis et al., 2012), while others have shown negative correlations, such that lower white matter integrity was associated with higher functional connectivity (Fling et al., 2012; Marstaller et al., 2015; Fjell et al., 2016; Yang et al., 2016). Still others have found no correlation (Hirsiger et al., 2016; Tsang et al., 2017), making it difficult to discern the relationship between these connectivity modalities. Some of these discrepancies could be related to differences in networks and white matter tracts examined across studies; future studies are needed to examine the impact of changes in coupling on cognitive and clinical outcomes. The fornix – salience network correlation, taken together with the significant effect of the salience network in our regression models, may suggest that the salience network is a key early functional connectivity marker to be further tracked during aging.

Elevated RD is often attributed to myelin loss (Alexander et al., 2011; Beaulieu, 2011; Tournier et al., 2011; Jones et al., 2013); therefore, a positive relationship between hippocampal cingulum RD and memory performance was not expected, nor was a negative relationship between salience network functional connectivity and memory performance. Nonetheless, the positive hippocampal cingulum – memory relationship was seen in our previous study in this cohort (for a longer discussion on the direction of this effect, see Alm et al., 2020), and two recent studies also reported negative correlations between functional connectivity and memory in non-demented older adults (Edde et al., 2020), as well as subjective cognitive decline and MCI patients (Xue et al., 2021). While it is true that RD generally increases (Lebel et al., 2012; Madden et al., 2012; Chen et al., 2013; Fjell et al., 2017; Ouyang et al., 2021) and functional connectivity generally decreases with age (Fjell et al., 2015, 2017; Sala-Llonch et al., 2015; Damoiseaux, 2017; Tsang et al., 2017), it is important to note that there are many exceptions to this general pattern (for reviews, see Ferreira and Busatto, 2013; Antonenko and Flöel, 2014). There have also been reports of increases and decreases in structural and functional connectivity in older adults within a single study, depending on the ROI/network (Madden et al., 2012; Tomasi and Volkow, 2012; Song et al., 2014; Ouyang et al., 2021; Xue et al., 2021; Pur et al., 2022) and whether within or between network connectivity is considered (Betzel et al., 2014; Song et al., 2014; Damoiseaux, 2017). In fact, Xue et al. (2021) found that specifically in the salience network, the direction of the functional connectivity – memory relationship in subjective cognitive decline and MCI patients depended on which salience network nodes were examined. Right anterior insula – left middle temporal gyrus functional connectivity was positively associated with memory, while right anterior insula – right superior temporal gyrus and right anterior insula – right hippocampus functional connectivity were negatively associated with memory. Thus, it is an oversimplification to assume that lower RD and higher functional connectivity should always be considered *better*, and therefore associated with higher memory scores. The underlying mechanisms are more complex, and the relationships with aging and cognition likely depend on a number of factors, such as the shape and length of connections and the specific ROIs.

Several limitations should be considered in the present investigation. First, this study was cross-sectional in nature. Since longitudinal neuroimaging data collection is underway for the BIOCARD study, future analyses will enable tracking of changes in structural and functional connectivity over time, as well as the examination of how connectivity changes may relate to longitudinal decline in memory performance. It should also be noted that the generalizability of these findings may be limited by BIOCARD cohort characteristics, including that participants were primarily white and highly educated. One technical limitation of diffusion imaging is that ROI-based analyses of small white matter tracts, such as the fornix, are particularly prone to signal contamination due to partial volume effects that can arise in regions within close proximity to CSF (Vos et al., 2011; Metzler-Baddeley et al., 2012). However, it is known that volume reductions due to atrophy exacerbate partial volume effects, creating a concern mostly in study samples with known atrophy, such as MCI or AD dementia patients (Oishi and Lyketsos, 2014). Future studies should take advantage of recent methodological advancements in diffusion imaging, including multi-shell acquisition and higher *b*-values, to further mitigate partial volume effects. Finally, the structural and functional connectivity measures used in this study are qualitatively different, complicating direct comparisons between the two modalities. Madden et al. (2020) argue that these differences in measurement properties may contribute to inconsistencies in past findings and suggest that graph theory measures may provide an approach to bring the different modalities into the same measurement framework.

## Conclusion

The current findings highlight the importance of multimodal approaches when considering neuroimaging-related markers of individual differences in episodic memory function among older adults. In this study, both structural and functional connectivity modalities uniquely contributed to explained variance in episodic memory performance within cognitively normal older adults. Furthermore, the combination of structural and functional connectivity markers best accounted for individual variability in episodic memory, suggesting meaningful information was gained by examining both connectivity modalities. Future studies will benefit from adopting a multimodal approach targeting these connectivity measures as critical markers of potential brain changes that may precede subsequent cognitive decline and pathological aging.

## Data Availability Statement

The raw data supporting the conclusions of this article will be made available by the authors upon request, without undue reservation.

## Ethics Statement

The studies involving human participants were reviewed and approved by the Johns Hopkins University Institutional Review Board. The patients/participants provided their written informed consent to participate in this study.

## Author Contributions

KA and AB conceptualized the study, generated the hypotheses, and acquired funding. KA curated the study sample data, performed the statistical analyses, generated figures and tables, and wrote the first draft of the manuscript. AS, CP, and MA oversaw data acquisition and curation, advised on study design and statistical analyses, provided resources and project administration, acquired funding, and revised and edited the manuscript. AF and SM oversaw data collection and analysis for diffusion imaging data. XH and HL oversaw data collection and analysis for resting state fMRI data. AM performed lumbar punctures and oversaw data collection and analysis of CSF data. AB advised on study design and statistical analyses, and revised and edited the manuscript. MA and AB supervised the project. All authors contributed to the manuscript and approved the submitted version.

## Conflict of Interest

AM receives research support from Fujirebio Diagnostics Ltd. SM is part owner and CEO of “AnatomyWorks.” This arrangement is being managed by Johns Hopkins University in accordance with its conflict of interest policies. AB is an inventor on Johns Hopkins University intellectual property with patents pending and licensed to AgeneBio. AB’s role in the current study was in compliance with the conflict of interest policies of the Johns Hopkins University School of Medicine. The remaining authors declare that the research was conducted in the absence of any commercial or financial relationships that could be construed as a potential conflict of interest.

## Publisher’s Note

All claims expressed in this article are solely those of the authors and do not necessarily represent those of their affiliated organizations, or those of the publisher, the editors and the reviewers. Any product that may be evaluated in this article, or claim that may be made by its manufacturer, is not guaranteed or endorsed by the publisher.

## Footnotes

1
https://braingps.mricloud.org
